# Histological Aspects of Rhinosinusal Polyps

**DOI:** 10.1016/S1808-8694(15)31090-9

**Published:** 2015-10-19

**Authors:** Luciano Gustavo Ferreira Couto, Atílio Maximino Fernades, Daniel Ferracioli Brandão, Dalisio de Santi Neto, Fabiana Cardoso Pereira Valera, Wilma T Anselmo-Lima

**Affiliations:** 1 Otorhinolaryngologist. Graduate student (master's degree); 2 Doctor in otorhinolaryngology, Ribeirão Preto Medical School, São Paulo University. Regular physician of the Otorhinolaryngology Unit, São Jose do Rio Preto Medical School, FAMERP; 3 Master in pathology, Ribeirão Preto Medical School, São Paulo University. Assistant physician in the Pathology Department of the Clinical Hospital, Ribeirão Preto Medical School, São Paulo University; 4 Master in pathology. Professor in the Pathology Department, São Jose do Rio Preto Medical School, FAMERP; 5 Doctor in otorhinolaryngology, Ribeirão Preto Medical School, São Paulo University. Regular physician of the Ophthalmology, Otorhinolaryngology and Head & Neck Surgery Department, Ribeirão Preto Medical School, São Paulo University; 6 Livre docente habilitation professor of the Ophthalmology, Otorhinolaryngology and Head & Neck Surgery Department, Ribeirão Preto Medical School, São Paulo University. Associate professor of the Ophthalmology, Otorhinolaryngology and Head & Neck Surgery Department, Ribeirão Preto Medical School, São Paulo University. Ribeirão Preto Medical School, São Paulo University

**Keywords:** biopsy, histological classification, light microscopy, nasal polyp

## Abstract

Contemporary cohort cross-sectional study. Introduction: Despite its importance for an accurate diagnosis, histology differences among nasal polyps and its clinical implications are rarely reported in the literature. The existing papers classify polyp samples without concern for prior treatments, which could influence the results attained.

**Aims:**

carry out a morphological study, through light microscopy, of nasal polyps' structural alterations in the absence of any type of prior treatment and histologically classify it in relation to studies published in the literature.

**Materials and Methods:**

We studied 89 patients with nasosinusal polyps without prior treatment. Polyp samples were collected by outpatient biopsy and analyzed through light microscopy after dyeing with hematoxylin-eosin.

**Results:**

Samples were classified in the following way: Edematous or eosinophilic polyp 65 cases (73%); fibro-inflammatory polyp: 16 cases (18%); Polyp with Sero-mucinose gland hyperplasia: 06 cases (6.7%) and polyp with stroma atypia: 2 cases (2.3%).

**Discussion:**

eosinophilic pattern prevailed in the patients with nasosinusal polyps of the population studied. This pattern is similar to the ones found in the major studies, which, however, do not mention prior treatment.

**Conclusion:**

after analyzing the polyps' histological characteristics, we noticed that the untreated polyps present a predominantly eosinophilic pattern.

## INTRODUCTION

Nasal polyposis is a chronic non-neoplastic inflammatory disease that is commonly encountered in clinical otorhinolaryngology.^1^ Its estimated incidence in the general population is 0.5 to 4%.^2^ Clinical manifestations include nasal obstruction, anterior and posterior rhinorrhea, anosmia and/or hyposmia, headaches and general malaise.^3^^,^^4^ Most of the polyps originate in the nasal mucosa of the middle meatus, although other ethmoidal sites may be involved.^5^

The etiology and pathogenesis of nasal polyposis has been studied since ancient times;^6^ however, in spite of the current understanding of this condition, particularly the role of inflammation, the mechanisms that cause nasal polyps remain unknown.^7^

Tos and Morgensen^8^ described rhinosinusal polyps histologically as having an edematous, predominantly eosinophil-infiltrated myxoid stroma covered by respiratory epithelium, which frequently presents hyperplasia or squamous metaplasia.

In the literature, however, there have been few studies on the histological differences among nasal polyps and possible clinical implications of such differences, which may be important for a precise diagnosis.^9^ The few existing papers on this topic have classified polyp samples collected in endoscopic surgery, but with little concern about the influence of previous topical or systemic therapy on the histology of these nasal polyps.^10^, ^11^, ^12^ Davidsson and Hellquist^10^ assessed 95 patients and found that an eosinophilic pattern was present in 83.6% of cases. In a study using optic and electronic microscopy we undertook in 2001,^12^ however, we found no eosinophilic polyps in 17 nasal polyps collected during surgery. On the other hand, the fibroinflammatory pattern that was found in most cases may have been due to two reasons: a difference in the predominant histological type, as occurs in the Asian population,^13^ or the influence of topical and systemic corticosteroids, which are routinely provided to our patients before surgery.

We decided, then, to undertake a morphological study using optic microscopy of structural alterations found in rhinosinusal polyposis samples that had not received any previous drug therapy, to classify these polyps histologically, and to correlate these findings with those encountered in the literature.

## PATIENTS AND METHOD

The study included 89 patients with rhinosinusal polyposis. All of these patients had not been treated with topical or systemic corticosteroids, antihistaminic drugs, antileukotrienes or antibiotics at least 30 (thirty) days before the biopsy.

After explanations and consent, patients underwent nasal polyp biopsies in an outpatient setting. Two or three Representative samples of lesions per patient were taken with Takahashi forceps. These samples were fixated in a formalin solution (10% formaldehyde), included in paraffin; 5μm sections were made, which were hematoxylin/eosin stained. Histological sections were made preferentially along the longitudinal axis of polyps for mounting the slides.

Polyp samples were exhaustively investigated using optic microscopy to characterize their morphological structure. This investigation was done at the pathology unit of our institution. Histological findings were grouped for classifying the polyps according to the following criteria:

1. Edematous or eosinophilic polyps: features stromal edema containing numerous eosinophils and mast cells, goblet cell hyperplasia in the respiratory epithelium and basal membrane thickening separating the epithelium from the edematous stroma.
2. Fibroinflammatory polyps: features a marked inflammatory infiltrate containing mostly lymphocytes. Other features include lack of stromal edema and goblet cell hyperplasia.
3. Polyps with seromucinous gland hyperplasia: features numerous seromucinous glands and ductal structures in an edematous stroma.
4. Polyps with stromal atypia: its characteristic feature is the presence of bizarre and atypical stromal cells. Cells may be irregular and hyperchromatic.

The Research Ethics Committee analyzed and approved this study. The process number was 8484/2005.

## RESULTS

There were 89 patients with a clinical and eventually a histological diagnosis of rhinosinusal polyposis, of whom 56 were males and 33 were females. Patients were aged between 18 and 76 years, mean 48 years.

The 89 polyp samples were analyzed and classified according to the four histological patterns described above.

**Figure 1:**
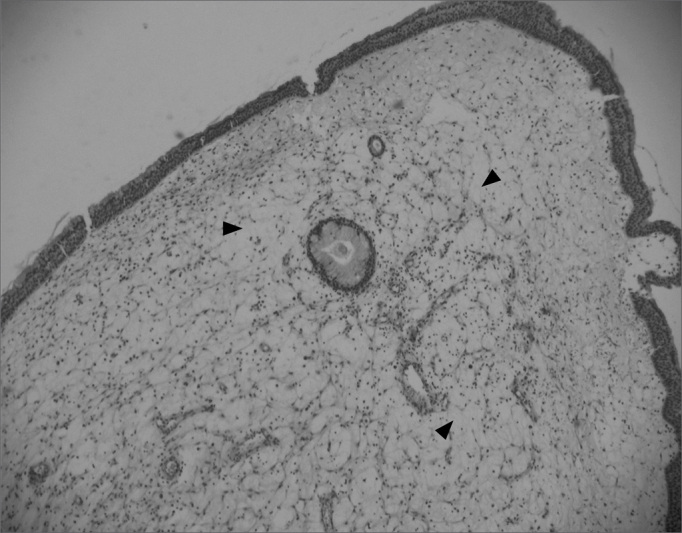
Edematous or eosinophilic polyp in a panoramic view. We can see the edema in the region of the stroma, with broad intercellular spaces (arrows). The stromal edema is partially filled by intercellular fluid, forming pseudocystic spaces (H&E × 40).

**Figure 2:**
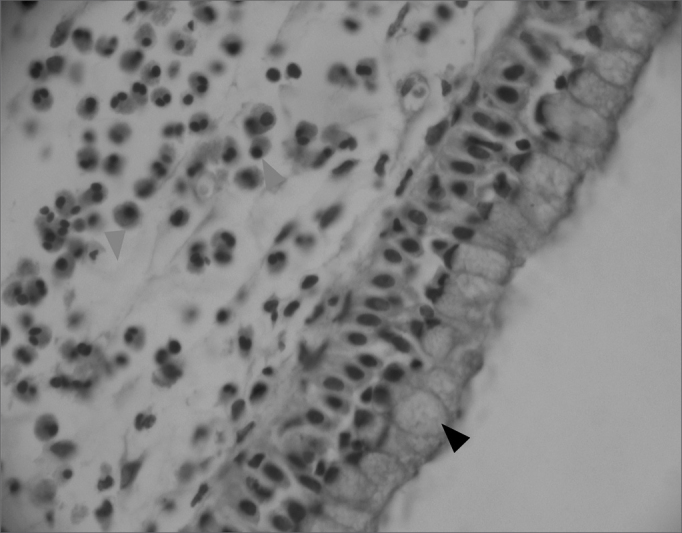
Edematous or eosinophilic polyp, with a marked hyperplasia of goblet cells in the epithelium (black arrow), broad intercellular spaces characterizing the stromal edema (green arrow) and a predominance of eosinophiles (blue arrow) among the inflammatory cells. (H&E × 400).

**Figure 3:**
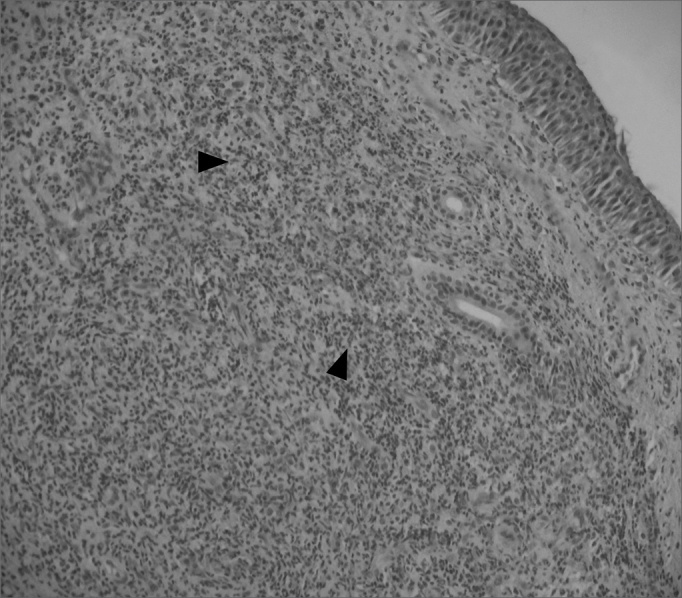
Fibro-inflammatory polyp, without stromal edema and goblet cells hyperplasia (its main characteristics). We also observe an intense inflammatory infiltrate in the stroma (arrows), and a predominance of lymphocytes (H&E × 40).

**Figure 4:**
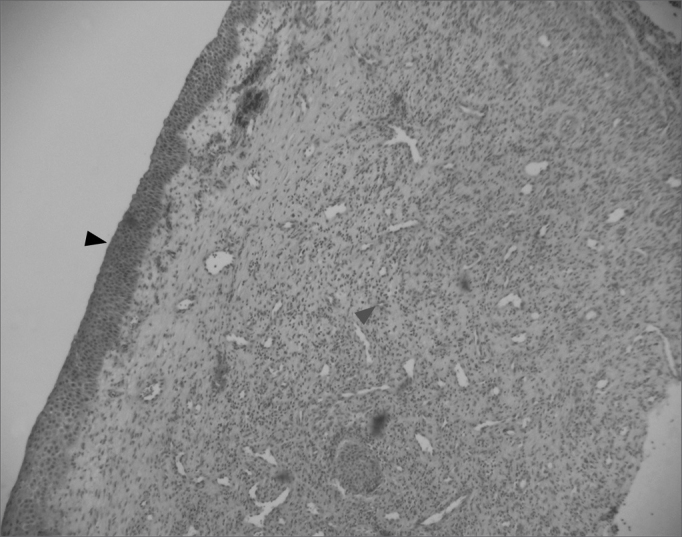
Fibro-inflammatory polyp with intense infiltrate of inflammatory cells in the stroma (green arrow) and epithelial squamous metaplasia (black arrow) (H&E × 40).

**Figura 5:**
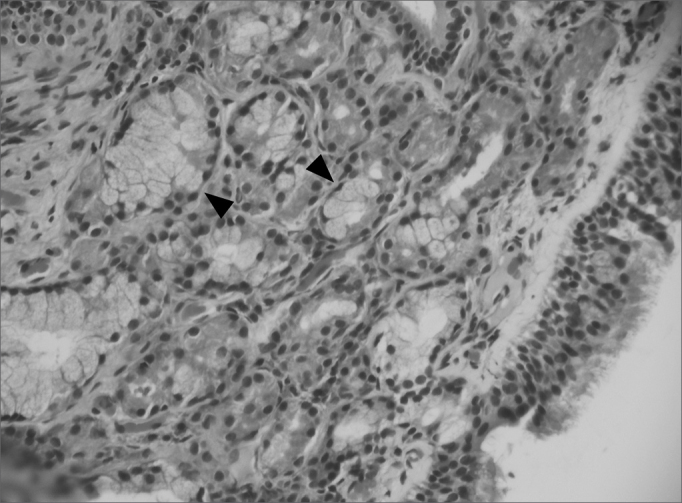
Polyp with hyperplasia of seromucinous glands. The abundance of glands (arrows) and duct structures is the characteristic that differentiates this polyp from the edematous or eosinophilic one (H&E × 200).

**Figura 6:**
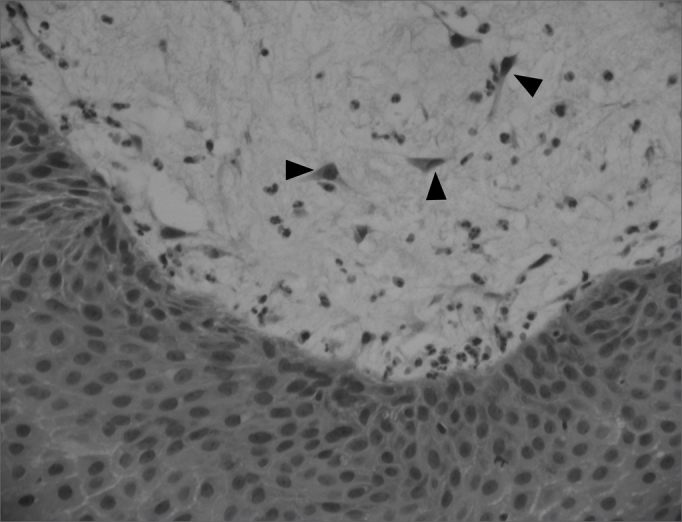
Polyp with stromal atypia and atypical cells (arrows). The cells tend to be hyperchromatic and with star-like cytoplasm projections. Occasionally the entire polyp may have these atypia. (H&E × 200).

Edematous or eosinophilic polyps predominated; these composed 65 cases, 73% of the total sample (Figure 1, Figure 2). The fibroinflammatory pattern was the second most frequent; these composed 16 samples, 18% of cases (Figure 3, Figure 4). Polyps with hyperplasia of seromucinous glands were less prevalent; these were found in 6 cases, 6.7% of the total (Figure 5). Polyps with stromal atypia were the rarest type among our sample; there were 2 such cases, 2.3% of the sample (Figure 6). The chart shows the results.

The chi-square method was used in the statistical analysis, for p<0.05.

**Chart 1:**
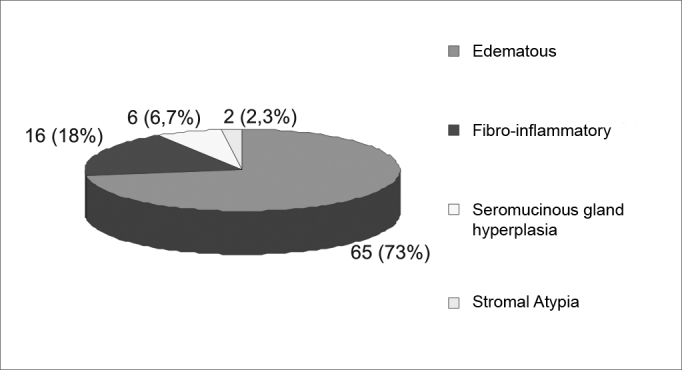
Histological Classification of the Nasal Polyp (number absolute of cases and percentage equivalent found).

## DISCUSSION

Studies on the pathophysiology of nasal polyps have aimed to understand this disease for more adequate treatment strategies. Topical nasal corticosteroids are the treatment of choice for nasal polyposis; their main action is to suppress inflammatory phases, and to reduce edema and the inflow of inflammatory cells into the respiratory mucosa. Other options are systemic corticosteroids and even antibiotics, such as the macrolides, which also have anti-inflammatory effects on nasal and sinusal polyps.^14^, ^15^, ^16^ Thus, the morphological structure of nasal polyps may undergo significant alterations due to medical treatment, particularly when topical and systemic corticosteroids are used. Based on this assumption, the purpose of our study was to analyze nasal polyp histology in patients that had not undergone drug therapy for polyps.

Kakoi and Hiraide^11^ (1987), in a series of 175 patients, subdivided polyps into:

• Edematous polyps: 60%
• Cystic or glandular polyps: 27%
• Fibrous polyps: 13%

Davidsson and Hellquist10 (1993) analyzed 95 patients and classified polyps histologically into four categories:

• Edematous, eosinophilic or “allergic,” polyps: 86.3%
• Fibroinflammatory polyps: 7.3%
• Polyps with seromucinous gland hyperplasia: 5.3%
• Polyps with stromal atypia: 1.1%

Hellquist (1996) analyzed in detail the histological differences between the polyps found in his first study.^9^ As a result, his paper became the main reference in the literature on the morphological classification of nasal polyposis. Our findings made it possible to group the polyps into histological patterns similar to those described by Hellquist. Notwithstanding our careful investigation, we found no case with enough novel features to deserve special attention as a new and previously unknown histological pattern of nasal polyps.

In the current study, we found that more than one of the histological patterns that we found may be present in any single polyp. This finding made it difficult, in some cases, to adequately characterize the sample. We therefore classified such polyps according to their most relevant features.

The histological classifications described above were based on polyps removed by functional endoscopic surgery. There were no references about any previous or concomitant use of medication. As surgery is indicated in those cases where medical therapy has failed or for the treatment of extensive polyposis, we assumed that there were no drug treatment-free cases before surgery or biopsy in those samples. Souza et al. (2001) found 14 cases of fibroinflammatory polyps, 1 case of polyp with gland hyperplasia and 2 cases that were named fibrotic polyps in a 17-case sample.^12^ The authors found no eosinophilic polyps, probably because all patients were undergoing topical and systemic corticosteroid treatment at the time of the biopsy. Although the sample was small in Souza et al.'s article, possibly the histological features of those polyps might have been altered by regular drug therapy, especially topical nasal corticosteroids; these drugs could have reduced the stromal edema and decreased the number of inflammatory cells in the stroma.

In our study, the eosinophilic pattern predominated in 73% of the sample, followed by the fibroinflammatory pattern, found in 18% of the sample. These number are similar to those published by Davidsson and Hellquist.^10^ Table 1 shows a comparison between various papers that have studied structural differences among rhinosinusal polyps. Polyps with stromal atypia were found in two cases. Macroscopically, they are similar to other polyps. Histologically, stromal myofibroblastic cells tend to exhibit hyperchromatic, enlarged nuclei and stellate cytoplasmatic projections. Lack of knowledge about this polyp and its histological features may lead the unwary pathologist to misdiagnose it as a malignant neoplasm. The main feature that differentiates a polyp with stromal atypia from a neoplasm is absence of mitoses.

**Table 1:** Histological classification of nasal polyps according to different studies in the literature.

Study × Histological Classification	Kakoi and Hiraide 1987^11^	Davidsson and Hellquist 1993^10^	Serra et al. 2001^17^	Couto et al. 2006
Edematous/Eosinophilic	105(60%)	82(86,3%)	00	65(73%)
Fibro-inflammatory	23(13%)	7(7,3%)	14(82,4%)	16(18%)
With hyperplasia of seromucous glands	47(27%)	5(5,3%)	1 (5,8%)	6(6,7%)
With Stromal Atypia	00	1(1,1%)	00	2(2,3%)
Fibrotic	00	00	2(11,8%)	00
Total	175	95	17	89

In comparing our findings with those of Davidsson & Hellquist (1993), a statistical analysis revealed a significant difference in the percentages of eosinophilic polyps (73% - Couto et al., 2007 < 86.3% - Davidsson & Hellquist, 1993; p<0.05) and of fibroinflammatory polyps (7.3% - Davidsson & Hellquist, 1993 < 18% - Couto et al., 2007; p<0.05).

Davidsson & Hellquist (1993) based their study on an analysis of the files of patients with operated nasosinusal polyposis. Their article contains no references about the preoperative use of medication; thus, no intrinsic cause-effect relation can be derived for these different percentages. Possible explanations for this difference might be the influence of population differences, and certain subjectivity in the histological analysis regardless of the examiner's experience or criteria, given that different histological features of polyps may be found in the same sample.

The current study appears to be the only one that is concerned with analyzing samples free from the influence of previous medical therapy. Our results revealed the same histological prevalence as that published by Hellquist in 1996, but with a few differences in proportion, such as a higher incidence of fibroinflammatory cases and a lower incidence of the edematous or eosinophilic type compared to that study.

## CONCLUSION

In this study we undertook a careful and detailed investigation of the histological features of nasal polyps in a representative sample of patients with no previous medical treatment of this condition.

There was a higher prevalence of edematous/eosinophilic polyps, followed by fibroinflammatory polyps, in the absence of previous medical treatment, which corroborates the more representative findings in the literature on this topic.
